# Does butein affect adipogenesis?

**DOI:** 10.1080/21623945.2019.1617619

**Published:** 2019-05-16

**Authors:** Bianca Hemmeryckx, Christine Vranckx, Dries Bauters, H. Roger Lijnen, Ilse Scroyen

**Affiliations:** Department Cardiovascular Sciences, Center for Molecular and Vascular Biology, KU Leuven, Leuven, Belgium

**Keywords:** Butein, ADAMTS5, adipogenesis, adipocyte differentiation, browning

## Abstract

Butein is a plant flavonoid chalcone, with presumed anti-adipogenic properties. It was reported to impair preadipocyte differentiation, limit adipose tissue (AT) development and enhance white AT browning in rodents. In this study, we investigated the hypothesis that these effects of butein may occur via reduction of ADAMTS5 (A Disintegrin And Metalloproteinase with ThromboSpondin motifs 5) expression. Murine 3T3-L1 or 3T3-F442A preadipocytes were differentiated into mature adipocytes in the presence of butein or vehicle. At regular time intervals RNA was collected for gene expression studies. Male hemizygous mice for Tg(Ucp1-luc2,-tdTomato)1Kajim (ThermoMouse) were exposed to butein or vehicle, after which ATs were analyzed for *Adamts5* and uncoupling protein-1 (*Ucp-1*) mRNA level changes. During preadipocyte differentiation, butein (25 – 50 mM) did not affect *Adamts5* or *Ucp-1* expression. Oil Red O analysis and monitoring of differentiation markers failed to demonstrate effects of butein on the differentiation extent. Furthermore, butein administration to the ThermoMouse (10 or 20 mg/kg, 4 days) or to the C57BL6/Rj mice (20 mg/kg, 4 weeks) did not enhance *Adamts5* or *Ucp-1* expression. Thus, we could not demonstrate marked effects of butein on the preadipocyte differentiation extent or AT development and browning, nor on *Adamts5* or *Ucp-1* gene expression during these processes.

## Introduction

Butein (3,4,2ʹ,4ʹ-tetrahydroxychalcone) is a flavonoid chalcone derived from various natural plants such as *Toxicodendron vernicifluum, Caragana jubata and Rhus verniciflua stokes*. It was reported to have anti-oxidant, anti-inflammatory, anti-cancer, anti-fibrogenic and anti-adipogenic activities [1–6]. Wang et al. [7] furthermore showed that butein has anti-inflammatory properties in adipocytes. Subsequently, it was shown that butein increased heme oxygenase-1 expression in adipocytes and in mice fed a high-fat diet (HFD), thereby preventing adipocyte hypertrophy and inflammation. *In vitro* studies with 3T3-L1 adipocytes indicated that this process was mediated by activation of the p38 mitogen-activated protein kinase/nuclear factor erythroid 2–related factor 2 pathways [8,9].

Butein was also shown to inhibit 3T3-L1 preadipocyte differentiation through the transforming growth factor-β pathway, followed by signal transducer and activator of transcription 3 and peroxisome proliferator-activated receptor (PPAR)γ signalling [6]. Interestingly, it was also reported that butein promotes browning of white adipose tissue (WAT) via PR domain zinc finger protein 4 induction [10]. In C3H10T1/2 adipocytes butein induced a robust induction of uncoupling protein-1 (*Ucp-1*) mRNA, a hallmark of browning [10]. In addition, in human chondrocytes, butein inhibited *Adamts5* (A Disintegrin And Metalloproteinase with ThromboSpondin motifs 5) gene expression [11].

Interestingly, we have previously shown that ADAMTS5 deficiency in mice kept on HFD is associated with reduced WAT development and enhanced browning of subcutaneous (SC) WAT, characterized by markedly enhanced UCP-1 expression [12–14]. Taken together, these studies raise the interesting hypothesis that the reported anti-adipogenic effect of butein and the enhanced browning of AT may be mediated by its inhibiting effect on ADAMTS5 expression. Therefore, we have in the present study evaluated a potential link between butein, ADAMTS5 and UCP-1.

## Materials and methods

### Animals

Breeding pairs of female noncarrier mice with male hemizygous mice for Tg(Ucp1-luc2,-tdTomato)1Kajim (ThermoMouse) in FVB/N background were purchased from The Jackson Laboratory (stock number 026690) and used to generate male ThermoMouse at the Animal Facility of the KU Leuven (Leuven, Belgium). All animals were housed in a temperature-controlled room with a 12-hour (h) light/dark cycle. They had *ad libitum* access to drinking water and were kept on standard chow (10.9 kJ/g, Ssniff). Body weight was measured of 10 8-week-old ThermoMouse mice, followed by a bioluminescence (BLI) scan at baseline (day 0). Subsequently, animals were divided in three groups: mice injected with vehicle (20% polyethylene glycol-4000 (PEG4000) in saline; n = 2), and mice injected intraperitoneally with butein (butein T, 10 or 20 mg/kg, n = 4) obtained from TCI Europe nv (B3803, batch number FGMKA-MB, purity on HPLC 99.8 area%). After 24 h (day 1), mice were again subjected to BLI. At day 3, mice were again injected and scanned after another 24 h (day 4). After scanning, mice were weighed and sacrificed by intraperitoneal injection of 60 mg kg-1 sodium pentobarbital (Dolethal, Abbott Laboratories). SC inguinal and intra-abdominal gonadal (GON) fat, and interscapular brown adipose tissue (BAT) were removed, weighed and snap-frozen in liquid nitrogen for RNA isolation and histology. Male C57BL6/Rj mice at an age of 8 weeks were purchased from Janvier Labs (Le Genest-Saint-Isle, France). At the age of 9 weeks, males were fasted for 6 h, and they were randomized into two groups based on an identical average body weight and fasting blood glucose level (measured at the tail using one drop of blood). All mice were exposed *ad libitum* to a methionine- and choline-deficient diet (MCD; 7 g/daily/mouse; MP Biomedicals, Illkirch Cedex, France) for 4 weeks, a diet that has been used by us in previous studies to induce steatohepatitis [15]. One group of mice (n = 5) received 20 mg/kg butein T once every two days (intraperitoneal injection) for 4 weeks long. Control mice (without butein; n = 5) received an intraperitoneal injection of the vehicle (saline with 20% PEG-4000% and 5% dimethylsulfoxide (DMSO)). Food intake and body weight were monitored weekly and the health of each animal daily, respectively. After 4 weeks, all animals were fasted for 6 h and blood glucose levels were measured via the tail. Animals were weighed and subsequently sacrificed with 60 mg/kg Dolethal (Abbott Laboratories). SC inguinal and intra-abdominal gonadal (GON) fat, and interscapular brown adipose tissue (BAT) were removed, weighed and snap-frozen in liquid nitrogen. SC fat was processed for RNA isolation to study the effect of butein on the expression of browning-associated markers. Liver was removed as well, weighed and processed for the measurement of triglyceride levels as described earlier [15]. All animal experiments were approved by the local Ethical Committee for Animal Experimentation (KU Leuven, P016/2013, P202/2017) and were performed in accordance with the NIH Guide for the Care and Use of Laboratory Animals (1996) and EU directive 2010/63/EU.

### *In vivo* bioluminescence imaging

To obtain *in vivo* bioluminescence measurements, mice with back and inguinal SC areas shaved, were anaesthetized using a mixture of 1.5–2% isoflurane in oxygen and were placed ventrally in a BLI camera (IVIS Spectrum; PerkinElmer) following the SC injection (neck area) of D-luciferin (150 mg/kg dissolved in phosphate-buffered saline (PBS), Promega BeNeLux). Luciferase activity was monitored in BAT using four consecutive scans with an acquisition time of 120 s starting immediately (i.e. within 10s) after D-luciferin administration until maximum signal intensity was reached. Subsequently, mice were placed dorsally and eight more consecutive scans of 120 s were acquired for SC fat. Settings were the following: binning: 8; F/stop: 1; emission filters: open; field of view: D. The BLI signal intensity was quantified using Living Image software v.4.2 (Perkin Elmer) and was reported as photon flux per second (p/s) for a region of interest of fixed size. The highest BLI signal intensity for each mouse in both areas was determined for baseline, day 1 and day 4. The photon flux per second for each tissue and each mouse at day 1 and day 4 was then normalized to the photon flux per second at baseline (p/s_day1,4_ divided by p/s_baseline_).

### *In vitro* differentiation of 3T3-L1 preadipocytes

Murine 3T3-L1 preadipocytes (p10 – p12) were grown in basal medium [Dulbecco’s Modified Eagle Medium (DMEM high glucose, Invitrogen) supplemented with 10% bovine calf serum iron supplemented (BCS; Hyclone Laboratories Inc.) and 1% Pen/Strep (Invitrogen; 10,000 units/ml penicillin and 10 mg/ml streptomycin)] in an atmosphere of 95% humidified air-5% CO_2_. Cells were passaged when preconfluent. To induce differentiation, cells were seeded at a density of 1 × 10^5^ cells/well in a 6-well plate and allowed to reach 100% confluency in basal medium (referred to as ‘day 0ʹ). At this time the basal medium was replaced by basal differentiation medium [DMEM high glucose supplemented with 10% fetal calf serum (FCS; Hyclone Laboratories Inc.) and 1% Pen/strep]. Furthermore, cells were at this time treated with 25 μM butein T or vehicle DMSO (dimethylsulfoxide). In another set of experiments, cells were treated with butein from a different source (butein A, 25 μM, ACM487525, batch number A17X03031, purity on HPLC 99.1 area%, ALFA Chemistry, Protheragen Inc.) or vehicle DMSO. As a positive control for the inhibition of preadipocyte differentiation [16], 3T3-L1 preadipocytes were exposed to 200 units bovine hyaluronidase type IV-S (100 U/ml, HYAL, H-4272, Sigma-Aldrich, Bornem, Belgium) or solvent H_2_O. After two days, differentiation was induced by replacing basal differentiation medium with induction medium [basal differentiation medium supplemented with 1 μg/ml insulin, 1 μM dexamethasone (DEX) and 0.5 mM methylisobutylxanthine (IBMX)]. At day 4 and thereafter, cultures were switched to differentiation medium (DMEM high glucose with 10% FBS, 1% Pen/strep, 1 μg/ml insulin), which was refreshed every two days. Compounds were also refreshed every other day. RNA was collected at different time points during the differentiation. On experimental day 10, the extent of differentiation was assessed by quantification of Oil Red O uptake by lipid-containing cells, and the viability of the cells was checked via exclusion of the Trypan Blue dye. At the end of the experiment, cells were washed with PBS, fixed in 1.5% glutaraldehyde in PBS for 5 min, stained for 3 h with a 0.2% Oil Red O solution (Sigma-Aldrich) at 37°C, washed and kept in tissue culture water. The stained fat droplets in the monolayer cells were visualized by light microscopy (Axiovert 200M inverted microscope, Zeiss, Jena, Germany) and photographed (AxioVision Rel. 4.8.2 software, Zeiss). For spectrophotometric quantification of lipid accumulation, the Oil Red O dye was extracted with isopropanol and the absorbance of the solution was read at 490 nm on an EL808 plate reader using Gen5 3.02 software (Bio-tek Instruments, Lucerne, Switzerland).

### *In vitro* differentiation of 3T3-F442A preadipocytes

Murine 3T3-F442A preadipocytes [17] at a low passage number (p5), a passage number between 13 and 14 or at a higher passage number (p16) were routinely grown at subconfluence in basal medium [DMEM High glucose (Invitrogen) supplemented with 10% BCS iron supplemented (Hyclone Laboratories Inc.) and 1% Pen/Strep (Invitrogen)] in an atmosphere of 95% humidified air-5% CO_2_. Cells were passaged when preconfluent. Cells were seeded at a density of 1 x 10^5^cells/well in a 6-well plate and grown to confluency (designated as ‘day 0ʹ) in basal medium. In a preliminary experiment to determine the optimal dose of butein, 3T3-F442A preadipocytes (p16) were treated with 5, 10, 20, 50 and 100 μM butein or vehicle DMSO. In another experiment, 3T3-F442A preadipocytes (both passage numbers) were treated with 25 μM and 50 μM butein T or vehicle DMSO. In another set of experiments, 3T3-F442A preadipocytes at p13-p14 were treated with 25 μM butein T or vehicle DMSO up to day 1 to investigate potential effects of butein on the early phase (0 – 24 h) of 3T3-F442A cell differentiation. Two sequential experiments with p5 cells were set up as follows: 50 μM butein or vehicle DMSO were added at two different time points, time of seeding (day −2) or day of confluency (day 0). In addition, a negative control for the differentiation was included by not exposing the 3T3-F442A preadipocytes to induction and differentiation medium (Blank Not dif.) versus cells that received both media (Blank). After two days, cells were induced to differentiate for 48 h with induction medium [DMEM high glucose supplemented with 10% FBS, 1% Pen/strep, 17.44 nM insulin, 2 nM tri-iodothyronine (T3), 100 nM DEX and 100 μM IBMX]. At day 4 and thereafter, cultures were switched to differentiation medium (DMEM high glucose with 10% FBS, 1% Pen/strep, 17.44 nM insulin and 2 nM T3), which was refreshed every two days. At regular time points during the differentiation, cell lysates were prepared for RNA extraction. On an experimental day 1 (24 h; p13-p14 cells), day 10 (p13-p14 cells, p5 cells) or day 12 (p16 cells), the extent of differentiation was assessed by quantification of Oil Red O uptake by lipid-containing cells and the viability was checked as described above.

### Gene expression analysis

The protocols for the DNA-free total RNA and cDNA preparation from cells and tissues have been described earlier [18]. Quantitative real-time PCR was performed in the ABI 7500 Fast Sequence detector using the TaqMan® Fast Universal PCR Master Mix and the following TaqMan® Gene Expression Assays (Applied Biosystems, Foster City, CA, USA): *Adamts5* (Mm00478620_m1), peroxisome proliferator-activated receptor (*Ppar)γ* (Mm0044940_m1), preadipocyte factor 1 (*Pref-1*, Mm00494477_m1), and *Ucp-1* (Mm01244861_m1). The expression of adipocyte protein 2 (*Ap2*; also known as fatty acid binding protein 4 or *Fabp4*) was determined with the primers and 6-carboxy-fluorescein (FAM) labelled probes reported elsewhere [19]. Fold differences in gene expression were calculated with the ΔΔCt method, using *β-actin* (Mm 01205647_g1) as a housekeeping gene.

### Adipocyte size analysis

Frozen SC and GON adipose tissues were fixed for 48 h in 4% formaldehyde, washed 3x with PBS, were immersed in 70% ethanol and processed to obtain paraffin-embedded samples. Sections of 10 μm from both tissues were stained with haematoxylin and eosin to identify individual adipocytes. Bright field images of stained sections were taken using an Axiovert 200M inverted microscope (Zeiss), the camera HRm (Zeiss) and the Axiovision Rel. 4.8.2. software (Zeiss) at a 200 x magnifications. The individual adipocyte size (square area) was determined for SC (number of images analysed: 9.0 ± 0.52; number of adipocytes analysed: 646 ± 8.1) and GON (number of images analysed: 12 ± 0.54; number of adipocytes analysed: 425 ± 5.7) adipose tissues by delineation of the circumference of the individual adipocytes using the NIH Image J software. For each image, the sizes of the individual adipocytes were sorted from the smallest to the largest value. Subsequently, the total number of adipocytes, the mean adipocyte size and the number of adipocytes in each adipocyte range (SC & GON: 0–500, 500–1,000, 1,000–1,500, 1,500–2,000, 2,000–2,500, 2,500–3,000, 3,000–8,000; GON: 3,000–3,500, 3,500–4,000, 4,000–12,000) was determined. The adipocyte distribution was calculated for each adipocyte size range by dividing the number of individual adipocytes in that range by the total adipocyte count and subsequently multiplying this obtained value by 100. For each animal, an average was obtained from the average adipocyte size and the adipocyte distribution for all images that were analysed for that particular animal. Subsequently, per group the values for both parameters for all animals in that group were averaged again.

### Western blot analysis

SC fat and BAT samples were homogenized with a FastPrep Ribolyser (MP Biomedicals, Elsene, Belgium) in 10 mM Na phosphate, pH 7.2, 150 mM NaCl, 1% Triton, 0.1% sodium dodecyl sulphate, 0.5% Na deoxycholate, 0.2% NaN_3_, containing a protease inhibitor cocktail (Thermo Fisher Scientific, Rockford, IL, USA). Protein concentrations were determined with the BCA protein assay (Thermo Fisher Scientific). An equal amount of protein (50 μg for SC fat and 10 µg for BAT) was loaded in each well of a 10% Bis-Tris NUPAGE gel (Thermo Fisher Scientific). Gels were transferred onto a 0.45 μm nitrocellulose membrane and blocked in 5% nonfat dry milk (Bio-Rad Laboratories N.V., Nazareth Eke, Belgium) in 10 mM Tris–HCl buffer containing 150 mM NaCl and 0.05% Tween 20 at pH 7.6 (TBST) for 2–3 h. Subsequently, membranes were cut into two pieces for the PGC-1α (peroxisome proliferator-activated receptor γ coactivator-1α) blot or was uncut for the UCP-1 blot. Membranes were probed with the following primary antibodies (at 4°C overnight): polyclonal rabbit anti-mouse α-tubulin antibody (bottom blot, PA522060, 1/2000 diluted in TBST supplemented with 5% bovine serum albumin (BSA), Thermo Fisher Scientific), the rabbit polyclonal antibody to PGC-1α (upper blot, NBP104676, 1/500 diluted in TBST containing 5% nonfat dry milk; Novus Biologicals, Centennial, CO, USA) and the rabbit polyclonal antibody to UCP-1 (whole blot, U6382, 1/2000 diluted in TBST supplemented with 5% BSA, Sigma-Aldrich). Secondary antibodies were goat anti-rabbit horseradish peroxidase-conjugated antibodies (P0448, 1/2000, DAKO, Glostrup, Denmark) diluted in TBST containing 0.5% non-fat dry milk for 1 h at room temperature. Signals were detected with Enhanced Chemiluminescence (Super Signal West Femto Maximum Sensitivity Substrate, Thermo Fisher Scientific). The UCP-1 western blot was stripped using the Re-Blot Plus Strong Solution (2504, 1/10 in water, EMD Millipore, Burlington, MA, USA), reprobed with the rabbit polyclonal antibody to α-tubulin and signals were detected using the Enhanced Chemiluminescence solution as mentioned above. The intensity of the PGC-1α, UCP-1 and α-tubulin bands were quantified using the NIH Image J software.

### Statistical analysis

Data are expressed as means ± SEM. Differences between two groups were analysed with the non-parametric Mann-Whitney U test, compatible with small sample sizes. Analysis was done with Prism 7 (GraphPad Software Inc., San Diego, CA, USA). Two-sided values of p < 0.05 are considered statistically significant.

## Results

### No effect of butein on Adamts5 during preadipocyte differentiation

**Figure 1.**
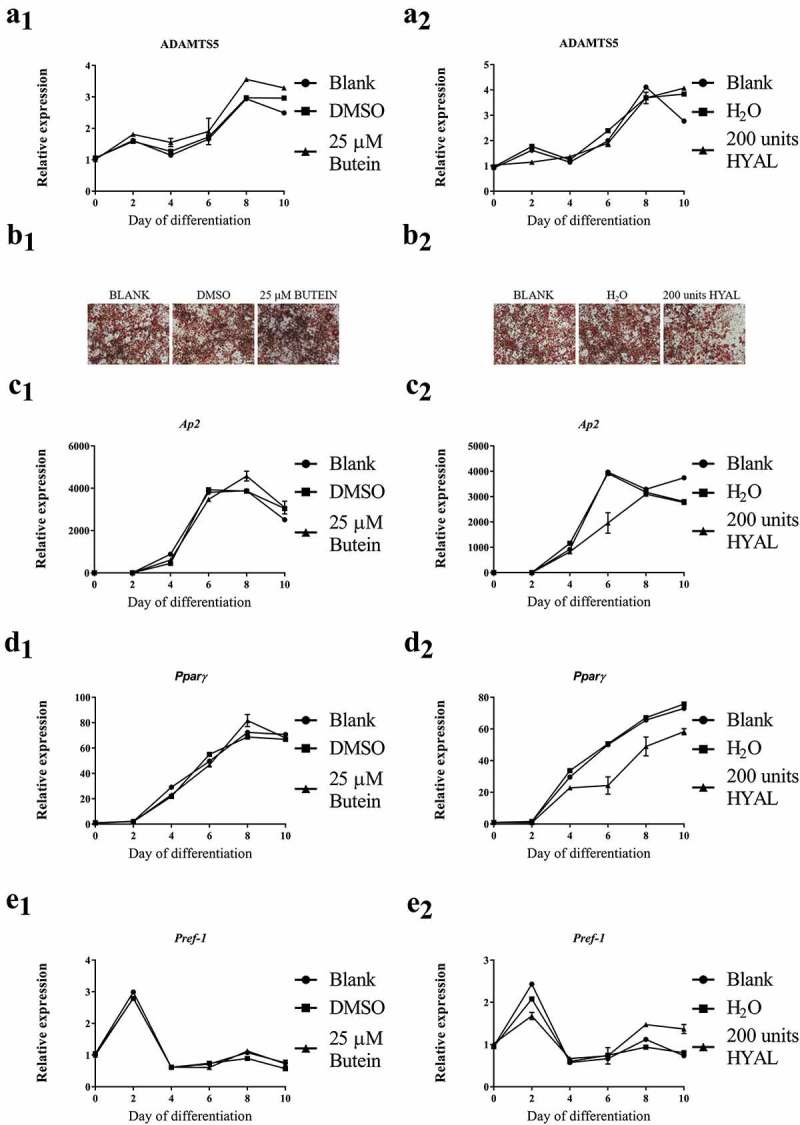
Effect of butein A (panels 1) or HYAL (panel 2) on differentiation of 3T3-L1 preadipocytes into mature adipocytes. Expression of *Adamts5* (panels a) and of the differentiation markers *Ap2* (panels c), *Pparγ* (panels d) and *Pref-1* (panels e) is shown as a function of time. Data are means ± SEM. Panels b represent Oil red O staining of differentiated 3T3-L1 preadipocytes (day 10) in the presence of butein, solvent DMSO or blank (panel b1) or in the presence of HYAL, solvent H_2_O or blank (panel b2). The scale bars correspond to 50 μm (magnification x100). Abbreviations: a disintegrin and metalloproteinase with thrombospondin motifs 5 (*Adamts5*), hyaluronic acid (HYAL), adipocyte protein 2 (*Ap2*), peroxisome proliferator-activated receptor γ (*Pparγ*), preadipocyte factor 1 (*Pref-1*) and dimethylsulfoxide (DMSO).

*Adamts5* expression during differentiation of 3T3-L1 preadipocytes into mature adipocytes increased with time, but was not affected by the addition of 25 μM butein A (Fig. 1A_1_). However, Oil Red O staining at day 10 did not indicate a significant effect of butein on the extent of differentiation (Fig. 1B_1_). Quantitative analysis of the staining confirmed these observations (OD^490 nm^ of 2.88 ± 0.041 for blank, 3.03 ± 0.074 for DMSO and 2.81 ± 0.092 for 25 μM butein). This was further confirmed by comparable expression of the differentiation markers *Ap2, Pparγ* and *Pref-1* (Fig. 1C_1_-E_1_). Trypan Blue staining showed that this dose of butein did not induce significant cell apoptosis (viability 87 ± 0% for blank, 89 ± 0% for DMSO and 89 ± 1% for butein).

In a parallel experiment, it was shown that the positive control HYAL (200 units per 2 ml) had no effect on *Adamts5* expression (Fig. 1A_2_), but did impair differentiation of the 3T3-L1 cells (Fig. 1B_2_). Oil Red O staining was reduced in the presence of HYAL (OD^490 nm^ of 2.34 ± 0.030 versus 3.17 ± 0.011 for blank and 2.95 ± 0.0010 for H_2_O solvent). At day 10 the normalized expression (to the expression at day 0) upon butein administration was lower for *Pparγ* and higher for *Pref-1*, as compared to blank or solvent (Fig. 1D_2_-E_2_). Cell viability was 91 ± 1.7% for HYAL as compared to 89 ± 0% for blank and 91 ± 1.7% for solvent.

To substantiate the lack of effect of butein, an independent experiment was performed with a second batch of butein of different origin (butein T, TCI Europe nv). During differentiation of the 3T3-L1 preadipocytes into mature adipocytes, the time course of mRNA expression of *Adamts5* as well as the differentiation markers *Ap2, Pparγ* and *Pref-1* was similar to that shown in Figure 1 (panels 1), and was not significantly affected by the addition of butein (data not shown). Quantitative analysis of Oil Red O stainings at day 10 (not shown) did not reveal a reducing effect of butein on differentiation (OD^490 nm^ of 2.77 ± 0.055 for DMSO versus 3.25 ± 0.073 for butein; p = 0.029).

**Figure 2.**
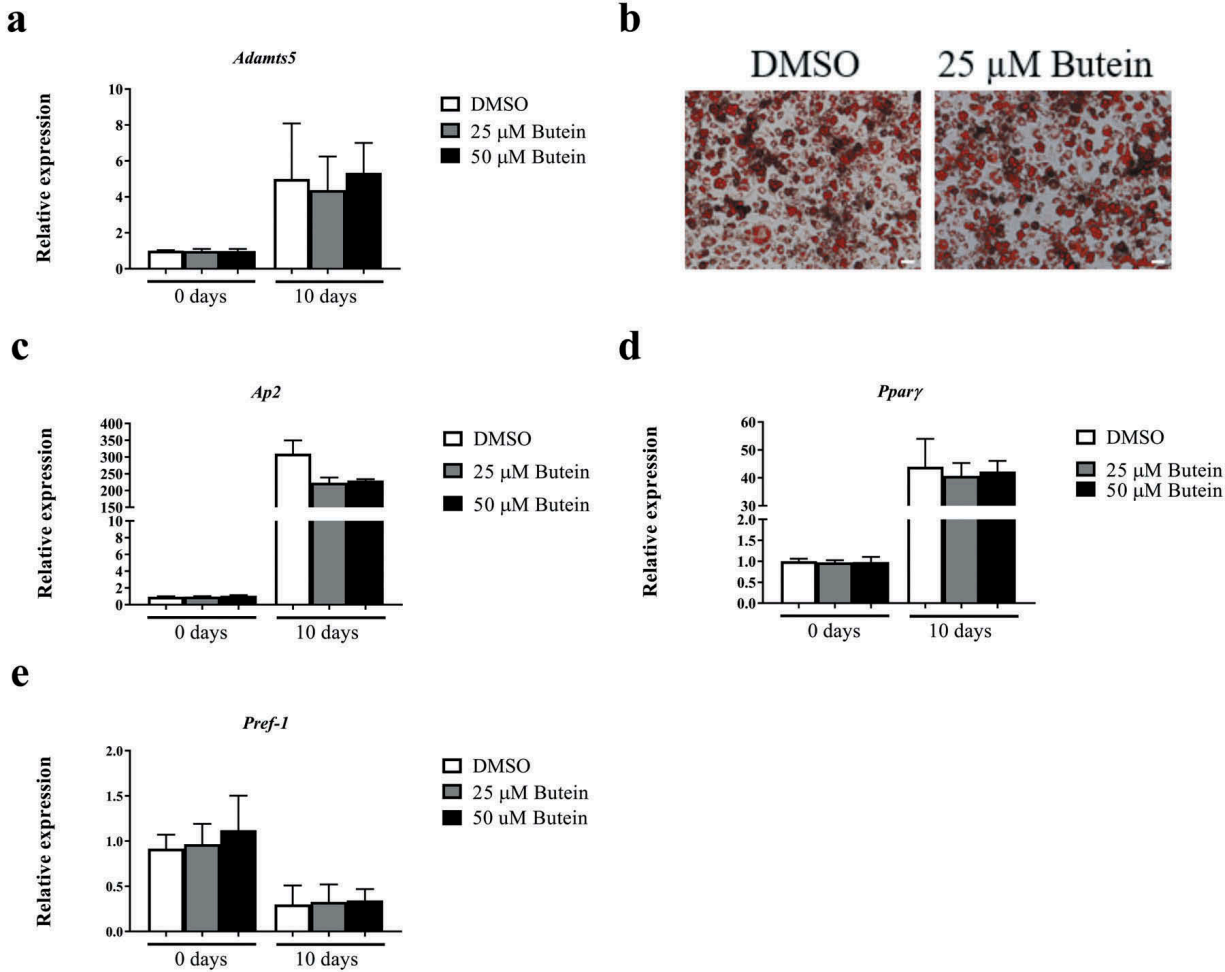
Effect of butein T on differentiation of 3T3-F442A preadipocytes into mature adipocytes. Expression of *Adamts5* (a) and of the differentiation markers *Ap2* (c), *Pparγ* (d) and *Pref-1* (e) is shown at the start and at day 10 of differentiation. Data are means ± SEM of 5 determinations in 2 independent experiments and are normalized to the blank value at day 0. Panel b shows Oil red O staining of differentiated 3T3-F442A preadipocytes (day 10) in the presence of butein (25 μM) or DMSO. The scale bars correspond to 50 μm (magnification x100). Abbreviations: a disintegrin and metalloproteinase with thrombospondin motifs 5 (*Adamts5*), adipocyte protein 2 (*Ap2*), peroxisome proliferator-activated receptor γ (*Pparγ*), preadipocyte factor 1 (*Pref-1*) and dimethylsulfoxide (DMSO).

To further study a potential effect of butein, *Adamts5* expression was also measured during differentiation of 3T3-F442A preadipocytes into mature adipocytes. After differentiation for 10 days, *Adamts5* expression was enhanced, but was not different for DMSO or butein-treated cells (Figure 2(a)). Oil Red O staining (Figure 2(b)) confirmed effective differentiation, but did not show a marked effect of butein, as confirmed by quantitative analysis (OD^490 nm^ of 2.41 ± 0.14 for DMSO, 2.58 ± 0.18 for 25 μM butein, and 2.65 ± 0.15 for 50 μM butein). This was further supported by comparable mRNA expression of *Ap2* (Figure 2(c)), *Pparγ* (Figure 2(d)) and *Pref-1* (Figure 2(e)). Trypan Blue staining confirmed that these doses of butein did not induce marked cell apoptosis (6.1 ± 0% for DMSO, 9.0 ± 0% for 25 μM butein and 11.9 ± 0% for 50 μM butein).

**Figure 3.**
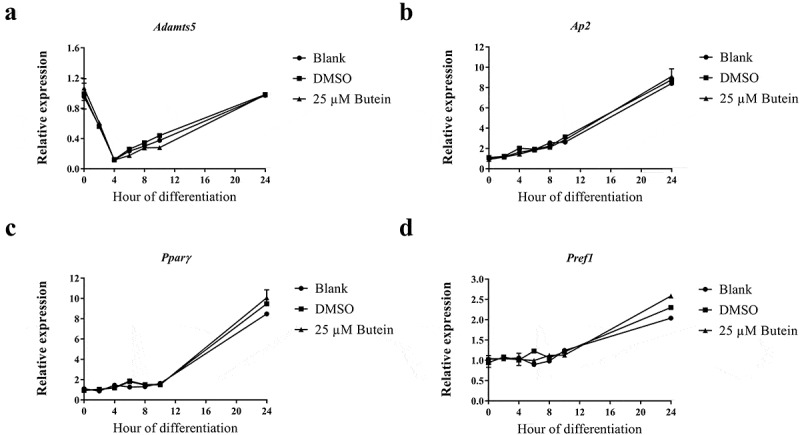
Effect of butein T on early phase differentiation of 3T3-F442A preadipocytes. The time course (0 – 24 h) of mRNA expression is shown for *Adamts5* (a), and for the differentiation markers *Ap2* (b), *Pparγ* (c) and *Pref-1* (d). Data are means ± SEM. Abbreviations: a disintegrin and metalloproteinase with thrombospondin motifs 5 (*Adamts5*), adipocyte protein 2 (*Ap2*), peroxisome proliferator-activated receptor γ (*Pparγ*), preadipocyte factor 1 (*Pref-1*) and dimethylsulphoxide (DMSO).

Subsequently, potential effects of butein (25 μM added to preconfluent cells) were investigated on the early phase (0–24 h) of 3T3-F442A cell differentiation. Under these conditions, *Adamts5* expression sharply declined within 4 h and then increased again, following a similar pattern for butein and vehicle (Figure 3(a)). Expression of the differentiation markers *Ap2* (Figure 3(b)), *Pparγ* (Figure 3(c)) and *Pref-1* (Figure 3(d)) was not different between blank, DMSO or butein.

**Figure 4.**
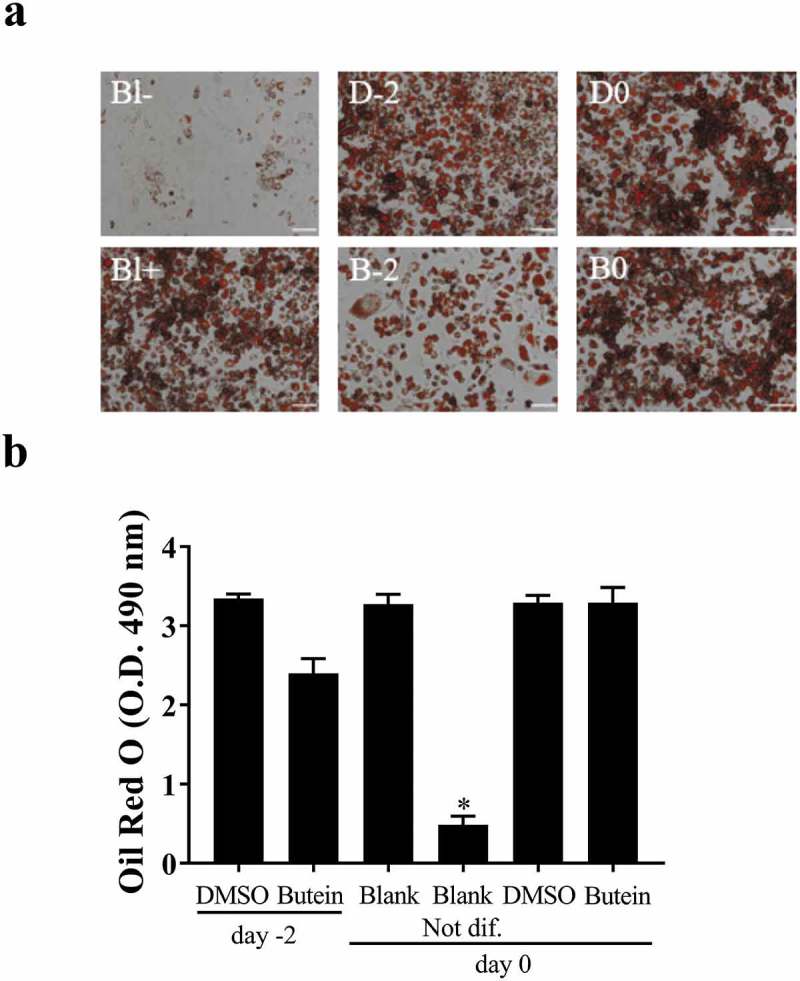
Effect of butein T on the triglyceride content of 3T3-F442A preadipocytes at day 10 of differentiation seeded at a lower passage. 3T3-F442A preadipocytes were differentiated in the presence of 50 μM butein or the vehicle DMSO starting at two different time points: day −2 (time of seeding the cells; butein-2 or B-2 versus DMSO-2 or D-2) or day 0 (time of confluency; butein or B0 versus DMSO or D0). Furthermore, 3T3-F442A preadipocytes were differentiated with (Blank or Bl+) or without the addition of induction and differentiation medium (Blank Not. dif. or Bl-). (a) Oil Red O staining. The scale bars correspond to 100 μm (magnification x100). (b) Quantification of Oil Red O staining. Data are means ± SEM of 3–4 measurements. * p < 0.05 versus Blank cells according to the Mann-Whitney U test.

**Figure 5.**
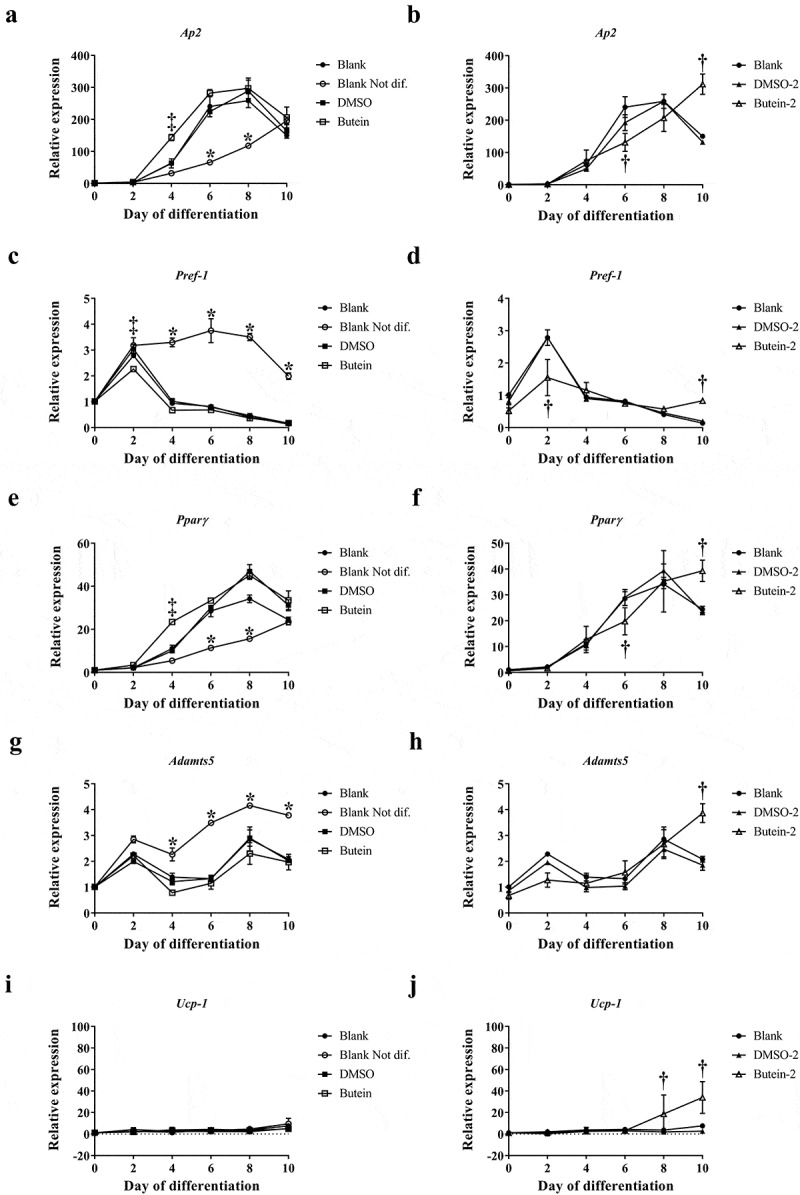
Effect of butein T on the differentiation of 3T3-F442A preadipocytes at a lower passage. 3T3-F442A preadipocytes were differentiated in the presence of 50 μM butein or the vehicle DMSO starting at two different time points: day −2 (time of seeding the cells; butein-2 versus DMSO-2; panels right) or day 0 (time of confluency; butein versus DMSO; panels left). Furthermore, 3T3-F442A preadipocytes were differentiated with (Blank) or without the addition of induction and differentiation medium (Blank not. dif.; panels left). (a–j) Gene expression analysis at day 10 of differentiation of the adipogenic markers *Ap2* (a–b), *Pref-1* (c–d), and *Pparγ* (e–f), and of *Adamts5* (g–h), and *Ucp-1* (i–j). Data are means ± SEM of 2–6 measurements. * p < 0.05 versus Blank cells; ^†^ p < 0.05 versus DMSO-2-treated cells and ^‡^ p < 0.05 versus DMSO-treated cells according to a two-way ANOVA with Bonferoni multiple comparison correction. Abbreviations: dimethylsulfoxide (DMSO); peroxisome proliferator-activated receptor γ (*Pparγ*); adipocyte protein 2 (*aP2*); preadipocyte factor-1 (*Pref-1)*; a disintegrin and metalloproteinase with thrombospondin type 1 motifs, member 4/5 (*Adamts4/5*) and uncoupling protein-1 (*Ucp-1*).

Interestingly, using an (older) batch of 3T3-F442A preadipocytes we initially determined the optimal dose of butein T by treating these cells with 0, 5, 10, 20, 50 and 100 μM butein T or vehicle DMSO. When 3T3-F442A cells were treated with 50 and 100 μM butein T, an increase in floating apoptotic cells was noticed as of day 2 and 4 of butein T treatment (data not shown). At day 12 of differentiation, differentiation was inhibited by butein at a concentration of 20 μM or higher (50 μM) as triglyceride content illustrated by the Oil Red O staining (Fig. S1(a)) and expression of the adipogenic markers Pparγ (Fig. S1(b)) and Lpl (Fig. S1(c)) were decreased in butein-treated versus DMSO-treated cells. Furthermore, at these concentrations, butein T also downregulated the expression of both *Adamts* (Fig. S1(d,e)) members, but not of *Ucp-1* (Fig. S1(f)). In a follow-up experiment we observed that upon addition of butein T, differentiation was indeed markedly impaired as shown by Oil Red O staining (OD^490 nm^ of 0.80 ± 0.011 at 25 μM and of 0.16 ± 0.019 at 50 μM butein T, as compared to 1.43 ± 0.028 for DMSO; p = 0.029). This was associated with a reduced normalized *Adamts5* mRNA expression (0.5 ± 0.019 at 50 μM butein versus 4.3 ± 0.23 with DMSO) relative to the mRNA expression level at day 0 of differentiation for the DMSO control. This could, however, be explained by extensive cell apoptosis during the differentiation period (14.6–34.8%). The effect of 50 μM butein T was also investigated in 3T3-F442A preadipocytes used at a low passage number (p5). Butein was applied at two different time points to analyse its effect on cell growth (day −2 or time of seeding the cells) and on cell differentiation (day 0 or time of confluency). When butein was added to the 3T3-F442A cells at day −2, less cells covered the surface of the well at day 2 of the differentiation (approximately 50%) and these cells never formed a confluent monolayer in comparison to the vehicle- or DMSO-2-treated cells. Furthermore, triglyceride content at day 10 of the differentiation (Figure 4(a,b)) tended to decrease in butein-2- versus DMSO-2-treated cells. Expression analysis of the adipogenic markers *Ap2* and *Pparγ* (Figure 5(b,f)) illustrated a slower increase in the expression of these markers in butein-2- versus DMSO-2-treated cells during the differentiation process. mRNA levels of *Pref-1* (Figure 5(d)) on the other hand were significantly downregulated at day 2, but were significantly higher at day 10 in butein-2- versus vehicle-treated cells. Expression of *Adamts5* (Figure 5(h)) and *Ucp-*1 (Figure 5(j)) were increased in butein-2- versus DMSO-2-treated cells, but only at the end of the differentiation (day 10 for *Adamts5* and day 8 and 10 for *Ucp-1*). However, when the butein has been added to the 3T3-F442A at day 0 when the cells form a confluent monolayer, butein does not impair differentiation of these cells as illustrated by the Oil Red O staining (Figure 4(a,b)) and the gene expression of the adipogenic markers (Figure 5(a,c,e)). At day 4 *Ap2* and *Pparγ* expression levels were significantly higher in butein- versus DMSO-treated cells, illustrating that butein may accelerate the differentiation process. Butein did not affect expression of *Adamts5* (Figure 5(g)) or *Ucp-1* (Figure 5(i)). Cells not exposed versus cells exposed to induction and differentiation medium (Blank Not dif. versus Blank) clearly showed that the differentiation process was impaired as Oil Red staining was significantly decreased (Figure 4(a,b)), and the mRNA levels of *Pref-1* (Figure 5(c)) remained elevated and the gene expression levels of Ap2 and *Pparγ* (Figure 5(a,e)) slowly increased during the differentiation process. Viability of all cells were above 90% for all groups.

**Figure 6.**
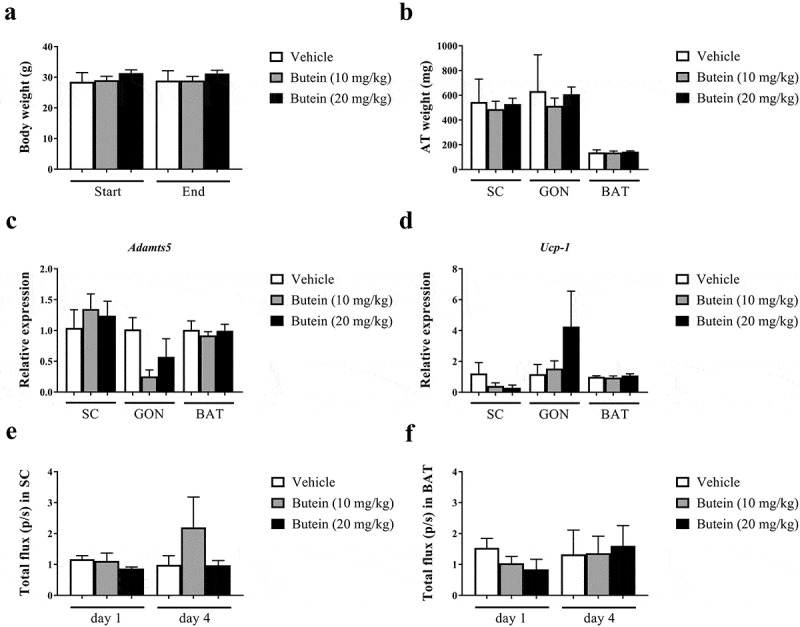
Effect of butein T on ADAMTS5 expression in the ThermoMouse. Body weight (a) and adipose tissue mass (b) (subcutaneous, SC; gonadal, GON; brown adipose tissue, BAT) are shown after 4 days treatment with butein (10 or 20 mg/kg) or vehicle. Expression of *Adamts5* (c) and *Ucp-1* (d) is shown in fat tissues isolated after 4 days. Data were obtained as cycle thresholds. For each animal and tissue, two duplicates of cDNA were applied and the results were averaged. Data were subsequently normalized to the housekeeping gene and to the calibrator vehicle-treated animals for each tissue. Bioluminescence analysis is shown at days 1 and 4 for SC fat (e) and BAT (f). Data are means ± SEM of 2 (vehicle) – 4 (butein both doses) experiments. Abbreviations: a disintegrin and metalloproteinase with thrombospondin motifs 5 (*Adamts5*) and uncoupling protein 1 (*Ucp-1).*

### No effect of butein T on ADAMTS5 in the Thermomouse

**Figure 7.**
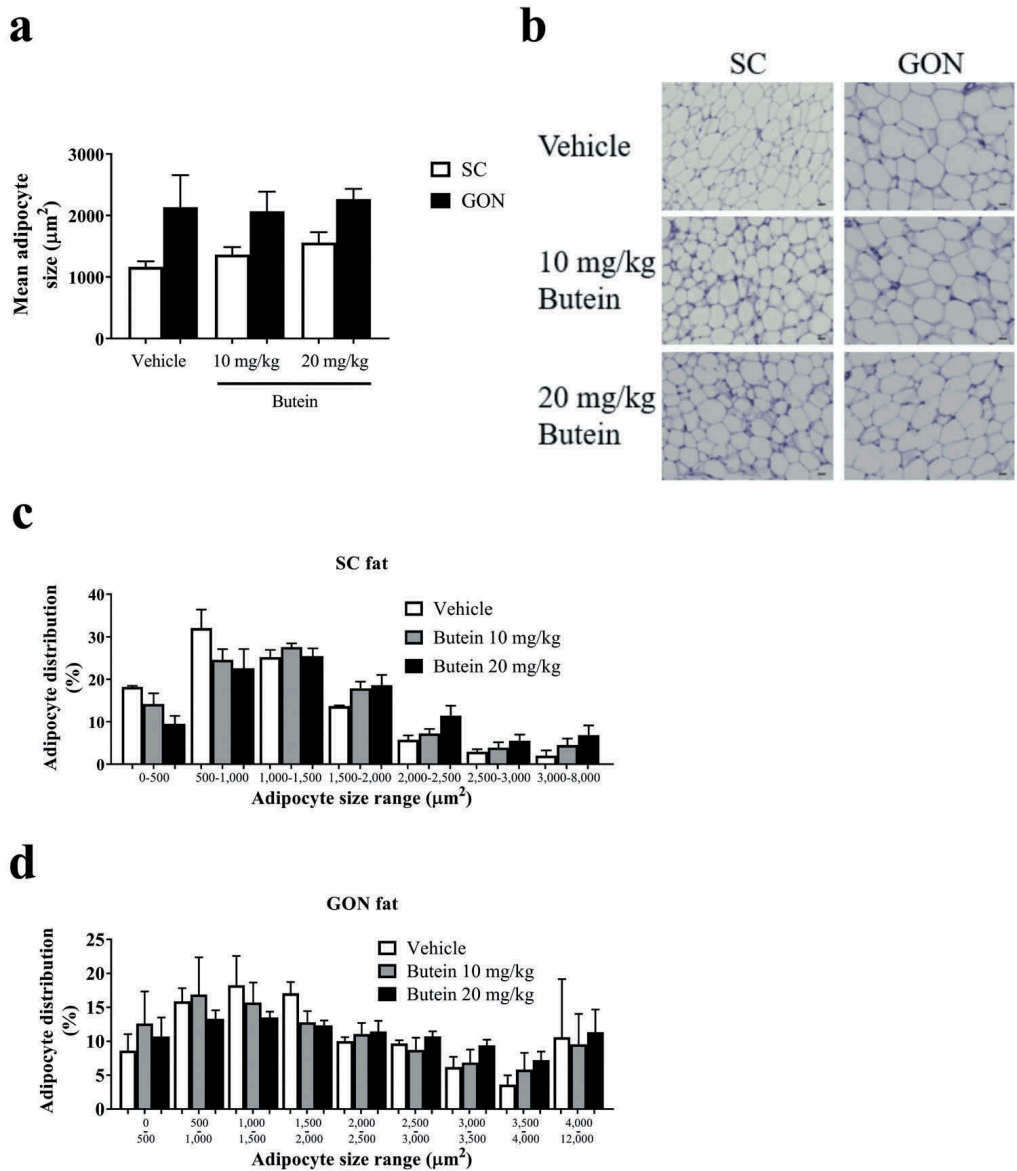
Effect of butein T on the mean adipocyte size and adipocyte distribution in the ThermoMouse. The mean adipocyte size (a) of subcutaneous (SC) and gonadal (GON) adipose tissues is shown after 4 days treatment with butein (10 or 20 mg/kg) or vehicle. (b) Paraffin sections of SC and GON adipose tissues obtained from these mice were stained with H&E to show individual adipocytes. Magnification: 200x. Scale bar: 20 μm. The distribution of individual adipocyte sizes is shown for SC (c) and GON (d) adipose tissues, respectively. Data are means ± SEM of n = 2 (vehicle) – 4 (butein both doses) experiments.

**Figure 8.**
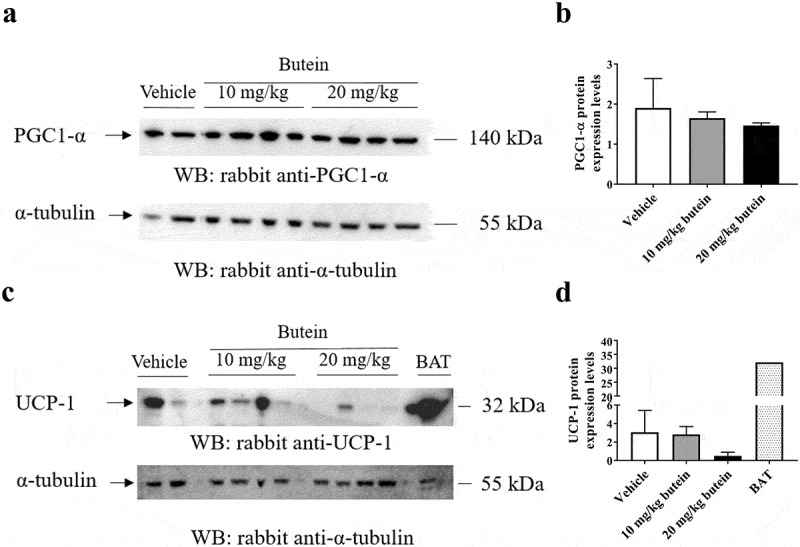
Effect of butein T on SC adipose tissue browning in the ThermoMouse. SC fat was obtained from ThermoMouse exposed for 4 days to butein (10 or 20 mg/kg; n = 4) or vehicle (n = 2). A BAT lysate of a ThermoMouse was used as a positive control for UCP-1 protein levels. Tissues were homogenized and protein lysates (50 µg for SC fat and 10 µg for BAT) were used in western blotting for PGC1-α (a) and UCP-1 (c). Panels (b) and (d) illustrate the densitometric quantitation of the PGC1-α and UCP-1 western blot, respectively, normalized for the levels of the loading control α-tubulin. Abbreviations: subcutaneous (SC); brown adipose tissue (BAT); peroxisome proliferator-activated receptor-γ coactivator 1-α (PGC1-α) and uncoupling protein-1 (UCP-1).

Administration of butein (10 or 20 mg/kg) over 4 days to the ThermoMouse did not affect total body weight nor SC, GON or BAT fat mass (Figure 6(a,b)). Analysis of the average adipocyte size (Figure 7(a,b)) or the distribution of the individual adipocyte size in SC (Figure 7(c)) and GON (Figure 7(d)) adipose tissues did not show an effect of butein exposure on these parameters. *Adamts5* or *Ucp-1* expression in fat tissues were not markedly affected by butein treatment (Figure 6(c,d)). However, a significant negative correlation was observed between *Ucp-1* and *Adamts5* expression, both in the vehicle-treated group (r = −1, p = 0.003) and in the butein-treated mice (both doses combined, r = −0.64, p = 0.0007). Bioluminescence analysis of SC fat or BAT confirmed that UCP-1 expression was not affected by butein administration (Figure 6(e,f)). In addition, PGC-1α (Figure 8(a,b)) and UCP-1 (Figure 8(c,d)) protein levels in SC fat were not different between butein- and vehicle-treated groups.

### No effect of butein T on AT development and hepatic triglyceride content in C57BL6/Rj mice

Body weight and blood glucose levels at the start of the study were similar between both groups (Table 1). After 4 weeks of MCD exposure, butein T did not affect body weight and blood glucose levels at the end of the study, body weight gain, food intake, and weight of the different organs (Table 1). *Ucp-*1 gene expression in SC fat was not different between the two groups (Butein: 1.5 ± 0.51; vehicle: 3.0 ± 1.2; p = 0.55), which illustrated that browning of SC fat was not affected by butein T at this dose and this experimental duration. Furthermore, hepatic triglyceride levels were similar as well between butein- and vehicle-treated animals (11 ± 2.8 mg/dl/mg tissue versus 17 ± 1.6 mg/dl/mg tissue; p = 0.15).

**Table 1.** Effect of butein T on body composition, food intake and fasting blood glucose levels in C57BL6/Rj mice exposed for 4 weeks to a methionine- and choline-deficient diet.

	Vehicle	Butein 20 mg/kg
Body weight (g)		
Start	21 ± 0.36	22 ± 0.51
End	15 ± 0.49	16 ± 0.44
Body weight gain (g)	–5.8 ± 0.29	–6.0 ± 0.26
Food intake (g/day)	2.2 ± 0.050	2.2 ± 0.25
Weight of organs (mg)		
SC fat	101 ± 10	102 ± 14
GON fat	59 ± 15	66 ± 12
Liver	453 ± 19	436 ± 12
BAT	42 ± 1.3	42 ± 1.9
Blood glucose levels (mg/dl)		
Start	160 ± 3.1	158 ± 4.8
End	98 ± 4.0	95 ± 6.4

Data are means ± SEM of n = 5 animals. Abbreviations: subcutaneous (SC); gonadal (GON), and brown adipose tissue (BAT).

## Discussion

AT inflammation and oxidative stress are key components in the development of obesity and insulin resistance. Butein was reported to have anti-inflammatory activity in adipocytes [7]. Thus, butein was shown to impair differentiation of 3T3-L1 preadipocytes into mature adipocytes, to limit AT development and to promote browning of WAT in rodents [6,8–10]. Other studies revealed an inhibitory effect of butein on ADAMTS5 expression in chondrocytes [11]. As we have previously shown that ADAMTS5 deficiency in mice limits WAT development and promotes browning of SC AT [12–14], we hypothesized that the reported effects of butein may be mediated via reduced repression of ADAMTS5. However, using two different-established preadipocyte lines (3T3-F442A and 3T3-L1) and butein of two different sources, we could not demonstrate an effect on *Adamts5* or *Ucp-1* expression, using butein concentrations comparable to other studies [6,8–10]. Surprisingly, in our experiments, butein did not significantly affect the extent of preadipocyte differentiation, as monitored by Oil Red O staining and determination of markers of differentiation. Since it was reported that early stage adipocyte differentiation is critical for the inhibitory action of butein [6], we added butein to the cells on day 0 of the 10-day differentiation procedure. Furthermore, we have shown that addition of butein to preconfluent preadipocytes has no effect on Adamts5, *Ap2, Pparγ* or *Pref-1* expression during the early phase (0 – 24 h). In our studies, cell apoptosis as monitored by Trypan blue staining was always less than 13% after 10 days in the presence of butein.

Interestingly, using 3T3-F442A cells at higher passage number (p16), butein markedly impaired *Adamts5* expression and reduced preadipocyte differentiation. This was however associated with pronounced apoptosis, which could explain the observed quantitative effects. Therefore, experiments were performed using 3T3-F442A cells at a low passage number (p5). The time of addition of the butein to the 3T3-F442A preadipocytes determined whether butein had an effect on the differentiation process. When butein was added at the time of seeding the cells (day −2), butein prevented the growth of the cells to a confluent monolayer, which is a prerequisite for a good differentiation experiment. However, when butein was added to the cells at day 0, a preconfluent stage where preadipocytes will form a confluent monolayer by dividing once more, butein initially accelerated the differentiation process (at day 4), but at day 10 no effect was noticed. Thus, the potential toxicity of butein may be due to the application of the compound in cells with a high passage number. The time of exposure of the cells to butein seems to be critical in the impaired effect of butein on *in vitro* preadipocyte differentiation.

It has been shown that butein-limited AT development in rodents [8]. We exposed the ThermoMouse and C57BL6/Rj mice to butein and found no effect of butein administration on AT development, which is in line with our *in vitro* adipocyte differentiation observations. Furthermore, liver triglycerides were not affected as well by butein treatment. It is, however, possible that a longer treatment and/or higher dose of butein is required to see an effect of butein on AT development. Indeed, Alshammari and colleagues (2018) exposed rats to the MCD diet for 6 weeks with or without butein treatment at a very high dose of 200 mg/kg [20]. The authors noticed a lower hepatic triglyceride content in butein-treated versus non-treated rats when they were exposed to the MCD diet. Although a study performed by Wang *et al*. [8] describes the exposure of 7-weeks-old male C57BL/6 mice to a normal diet or to a high-fat diet (60%) combined with or without a treatment with butein (ip injection 3x weekly, 10 mg/kg) for a duration of 3 weeks. Although the exposure time and concentration of butein was lower as compared to our study with C57BL6/Rj mice, the authors did find a reduction in epididymal fat mass and mean surface area of this adipose tissue in butein-treated animals on a HFD versus the HFD-fed control mice.

It was also reported that butein has the potential to enhance browning of WAT [10]. Increasing the thermogenic activity of adipocytes through browning of AT is considered an interesting approach to limit obesity and its related metabolic diseases [21–23]. As we have previously shown that ADAMTS5 deficiency in mice promotes browning of SC AT [13], we have investigated whether this reported effect of butein may be mediated via reduced expression of *Adamts5*. Therefore, we have used the ThermoMouse, an established *in vivo* model for rapid (within days) identification of modulators of *Ucp-1* expression. UCP-1 indeed is the hallmark of thermogenesis and energy dissipation, and is monitored in this model via luciferase activity [24]. However, administration of butein at doses used in other *in vivo* studies [8,25,26] did not affect *Ucp-1* expression as measured *in vivo* or in isolated adipose tissues. In addition, analysis of PGC-1α and UCP-1 protein levels in SC fat lysates showed a similar protein expression level of both proteins between vehicle- and butein-treated mice. A limitation of this *in vivo* study is that no appropriate control for the induction of browning of white adipose tissue in the ThermoMouse mice (either through a 4 days cold exposure or treatment with the β3-adrenergic receptor agonist CL-316,243) is lacking. In the C57BL6/Rj mice exposed for 4 weeks to 20 mg/kg butein and the MCD no effect of butein on SC *Ucp-*1 gene expression was observed as well. We could thus not confirm effects of butein on UCP-1 expression or browning of AT. Interestingly, in the ThermoMouse model *Ucp-1* expression correlated negatively with *Adamts5* expression, supporting the previously established interaction between both [13]. It is, however, possible that longer exposure times and higher doses of butein are needed to notice the induction of *Ucp-1* expression *in vivo*.

In summary, our *in vitro* studies with two different preparations of butein and two preadipocyte cell lines only confirmed an inhibitory role of butein on adipogenesis, when butein was added to growing preadipocytes and to cells with a high passage number. Our *in vivo* studies using the ThermoMouse and the MCD-fed C57BL6/J mice could not confirm an inhibitory effect of butein on browning of AT, which may be because the dose and duration of the treatment were not optimal.

## Supplementary Material

Supplemental Material
