# Supplementary material for: Does butein affect adipogenesis?
Source: Adipocyte. 2019 May 16;8(1):209–22. doi: 10.1080/21623945.2019.1617619 (PMC6768229; doi:10.1080/21623945.2019.1617619)
Supplement: Supplemental Material [file kadi-08-01-1617619-s001.zip › FigS1 caption.docx]

**Fig. S1.** Butein T dose-dependently inhibited 3T3-F442A preadipocyte differentiation. 3T3-F442A preadipocytes were differentiated in the presence of butein T at different concentrations or the vehicle DMSO. A) Quantification of Oil Red O staining at day 12 of differentiation. B-F) Gene expression analysis at day 12 of differentiation of the adipogenic markers *Pparγ* (B) and *Lpl* (C) and of *Adamts5* (D), *Ucp-1* (E) and *Adamts4*. Data are means ± SEM of 4 wells. * p < 0.05 versus DMSO-treated cells according to the non-parametric Mann-Whitney U test. Abbreviations: dimethylsulfoxide (DMSO); peroxisome proliferator-activated receptor γ (*Pparγ*); lipoprotein lipase (*Lpl*); a disintegrin and metalloproteinase with thrombospondin type 1 motifs, member 4 or 5 (*Adamts4/5*) and uncoupling protein-1 (*Ucp-1*).
